# Direct Synthesis of MoS_2_ Nanosheets in Reduced Graphene Oxide Nanoscroll for Enhanced Photodetection

**DOI:** 10.3390/nano12091581

**Published:** 2022-05-06

**Authors:** Zhikang Wu, Feifei Li, Xiya Li, Yang Yang, Xiao Huang, Hai Li

**Affiliations:** Key Laboratory of Flexible Electronics (KLOFE) & Institute of Advanced Materials (IAM), Nanjing Tech University (Nanjing Tech), 30 South Puzhu Road, Nanjing 211816, China; iamzkwu@njtech.edu.cn (Z.W.); lifeifei@njtech.edu.cn (F.L.); lixiya@njtech.edu.cn (X.L.); iamyyang@njtech.edu.cn (Y.Y.)

**Keywords:** ammonium tetrathiomolybdate, thermal annealing, MoS_2_@reduced graphene oxide nanoscroll, photosensitivity, photodetection

## Abstract

Due to their unique tubular and spiral structure, graphene and graphene oxide nanoscrolls (GONS) have shown extensive applications in various fields. However, it is still a challenge to improve the optoelectronic application of graphene and GONS because of the zero bandgap of graphene. Herein, ammonium tetrathiomolybdate ((NH_4_)_2_MoS_4_) was firstly wrapped into the ((NH_4_)_2_MoS_4_@GONS) by molecular combing the mixture of (NH_4_)_2_MoS_4_ and GO solution on hydrophobic substrate. After thermal annealing, the (NH_4_)_2_MoS_4_ and GO were converted to MoS_2_ nanosheets and reduced GO (RGO) simultaneously, and, thus, the MoS_2_@RGONS was obtained. Raman spectroscopy and high-resolution transmission electron microscopy were used to confirm the formation of MoS_2_ nanosheets among the RGONS. The amount of MoS_2_ wrapped in RGONS increased with the increasing height of GONS, which is confirmed by the atomic force microscopy and Raman spectroscopy. The as-prepared MoS_2_@RGONS showed much better photoresponse than the RGONS under visible light. The photocurrent-to-dark current ratios of photodetectors based on MoS_2_@RGONS are ~570, 360 and 140 under blue, red and green lasers, respectively, which are 81, 144 and 35 times of the photodetectors based on RGONS. Moreover, the MoS_2_@RGONS-based photodetector exhibited good power-dependent photoresponse. Our work indicates that the MoS_2_@RGONS is expected to be a promising material in the fields of optoelectronic devices and flexible electronics.

## 1. Introduction

By scrolling two-dimensional graphene nanosheet into a one-dimensional structure, a graphene nanoscroll (GNS) is formed with a tubular, spiral structure and open ends [1]. Due to the excellent properties resulting from its unique structure, graphene nanoscroll has attracted great attention in the fields of energy storage, sensors and flexible electronics [2,3,4,5]. In recent years, graphene oxide nanoscrolls (GONS) have been widely investigated instead of GNS because of the facile mass production of graphene oxide [2,6,7,8,9,10,11,12,13,14,15]. In order to further improve the performance of GONS, various functional nanomaterials were wrapped into GONS to extend their applications in supercapacitors, batteries and photocatalysts [16,17,18,19,20]. By encapsulating sulfur into GONS during the freeze-casting process, the as-prepared S/GONS was used as a good cathode material for a lithium-sulfur battery [21,22]. After Fe_2_O_3_ nanoparticles were wrapped into GONS as electrode materials by ultrasonication, a high volumetric energy density supercapacitor with quite good cycling stability was obtained [23]. Fe_1−x_S/Fe_3_O_4_ nanoparticles were also confined into GONSs and GO nanosheets by cold quenching and freeze drying, and the as-obtained composites were used as promising electrodes for a flexible lithium-ion battery [3].

It is well known that the application of graphene in high-performance photodetectors has been seriously hindered due to its low optical absorption ability [24]. To improve the photoresponse of graphene-based devices, a large number of functional nanomaterials have been integrated with graphene, including quantum dots [24], transition metal dichalcogenides [25,26,27], metallic nanostructures with plasmonic effects [28,29] and so on. Although the GONS has shown promising applications in the fields of energy storage, sensing and photocatalysis, it is still a challenge to apply the GONS as a high-performance photodetector. Previously, we have embedded chemical vapor deposition (CVD)-grown MoS_2_ nanoflakes into RGO nanoscrolls to improve the photodetection performance [25]. The photosensitivity of MoS_2_@RGO nanoscrolls increased by 20 times compared to the RGO nanosheet. However, it is difficult to further increase the amount of MoS_2_ grown on an RGO nanosheet by using the CVD method. Therefore, it is highly desirable to develop an alternative method to wrap a large amount of MoS_2_ into GONS and thus further enhance its photodetection performance.

In this work, a large amount of a precursor, ammonium tetrathiomolybdate ((NH_4_)_2_MoS_4_), was successfully wrapped into GONSs with length up to hundreds of micrometers by using the molecular combing method. After high temperature annealing, the (NH_4_)_2_MoS_4_ was in situ decomposed to MoS_2_ nanosheets, which were well encapsulated into the GONS. Meanwhile, the GONSs were simultaneously converted to reduced GONSs (RGONS). By optimizing precursor concentration and annealing temperature, high-quality MoS_2_@RGONS was facilely obtained with mass production. The optical microscopy (OM), atomic force microscopy (AFM), high-resolution transmission electron microscopy (HRTEM) and Raman spectroscopy were employed to demonstrate the uniform distribution of MoS_2_ nanosheets in RGONSs. The photodetectors based on MoS_2_@RGONSs showed photosensitivity two orders of magnitude higher than that of the RGONS under visible light. The MoS_2_@RGONSs-based photodetectors also exhibited good power dependent behavior. The improved performance could be attributed to the formation of multiple graphene/MoS_2_ interfaces in the scrolled structure of MoS_2_@RGONS, which increases the light absorption efficiency and enables the ultrafast charge transfer, resulting in a much higher photocurrent and photosensitivity. 

## 2. Experimental Section

### 2.1. Preparation of (NH_4_)_2_MoS_4_ Solution and Hydrophobic Substrate

Graphene oxide (GO) was synthesized by the modified Hummers method. A total of 0.038 g of (NH_4_)_2_MoS_4_ was ground to fine powder and dissolved in water to form aqueous solutions with concentration of 5, 10, 20, 30 and 50 mM. Thereafter, the (NH_4_)_2_MoS_4_ solution was treated by ultrasonication to ensure complete dissolution. Finally, the (NH_4_)_2_MoS_4_@GO solution was prepared by mixing 0.2 mg/mL GO and (NH_4_)_2_MoS_4_ solution with a volume ratio of 1:1. 

The preparation of hydrophobic substrate is a key step to form GO nanoscrolls by the molecular combing method [6,7,10,11]. Firstly, the cleaned 300 nm SiO_2_/Si substrate was immersed into a glass bottle containing a mixture of 8 mL toluene and 200 µL OTS (Octadecyltrimethoxysilane, purchased from Aladdin). Then, the glass bottle was sealed and heated at 60 °C for 24 h. After that, the SiO_2_/Si substrate was washed with ethanol and DI water three times. Therefore, the hydrophobic OTS-modified SiO_2_/Si substrate (OTS-SiO_2_/Si) was obtained. 

### 2.2. Preparation of (NH_4_)_2_MoS_4_@GONS and MoS_2_@RGONS

**Scheme 1:**
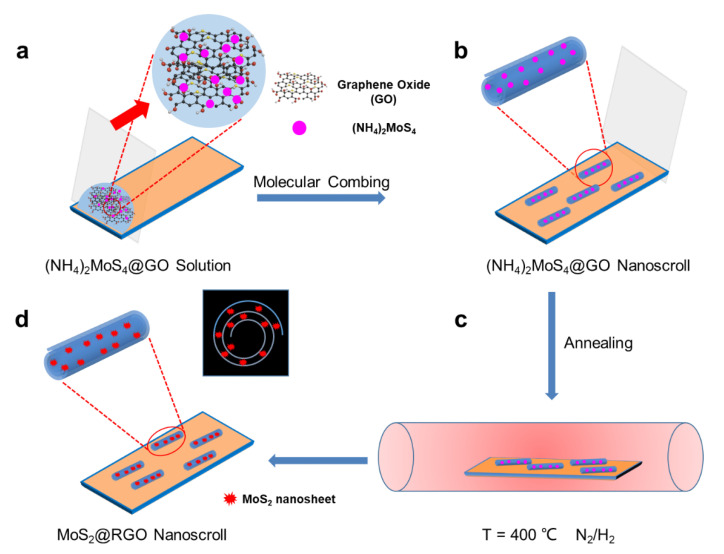
Schematic diagram of the preparation of (NH_4_)_2_MoS_4_@GO and MoS_2_@RGO nanoscrolls. (**a**) A drop of (NH_4_)_2_MoS_4_ and GO solution is dragged by the cover slip on the hydrophobic substrate. (**b**) The (NH_4_)_2_MoS_4_@GO nanoscroll is formed by molecular combing. (**c**) After the as-prepared (NH_4_)_2_MoS_4_@GO nanoscroll is treated at 400 °C in the N_2_/H_2_ environment, (NH_4_)_2_MoS_4_ is decomposed to MoS_2_ nanosheets and GO is reduced to RGO. (**d**) The as-obtained MoS_2_@RGO nanoscroll after high temperature annealing. The inset in the top right shows the cross-section structure of the MoS_2_@RGO nanoscroll.

The (NH_4_)_2_MoS_4_@GO nanoscrolls ((NH_4_)_2_MoS_4_@GONSs) were prepared by the molecular combing method [6], as shown in Scheme 1a,b. Firstly, 30 μL of the (NH_4_)_2_MoS_4_@GO solution was dropped onto the hydrophobic OTS-SiO_2_/Si substrate. A glass coverslip was then used to slowly drag the droplet from one end to the other end of the substrate. In this way, the (NH_4_)_2_MoS_4_@GONSs were formed on the OTS-SiO_2_/Si substrate. The MoS_2_@RGO nanoscrolls (MoS_2_@RGONSs) were prepared as shown in Scheme 1c,d. After the (NH_4_)_2_MoS_4_@GONSs were put into a tube furnace, a mixture gas of N_2_/H_2_ (80/40 sccm) was introduced as a protective gas. Then, the temperature of the furnace increased to 400 °C gradually, with a speed of 10 °C/min, and was kept for 60 min. During this period, (NH_4_)_2_MoS_4_ was decomposed to MoS_2_, while GO was reduced to RGO at the same time. Therefore, the MoS_2_@RGONSs were successfully obtained.

### 2.3. Characterizations

An optical microscope (ECLIPSE LV100ND, Nikon, Tokyo, Japan), AFM (Dimension ICON with Nanoscope V controller, Bruker, Billerica, MA, USA) and TEM (JEM-2100F JEOL, Tokyo, Japan) were used to characterize the as-prepared (NH_4_)_2_MoS_4_@GONS and MoS_2_@RGONS. In addition, Raman spectroscopy and Raman mapping of the as-prepared (NH_4_)_2_MoS_4_@GONS and MoS_2_@RGONS were tested on a LabRAM HR Evolution Raman spectrometer (Horiba Jobin Yvon, Palaiseau, France) with a 532 nm laser focused through a 100× objective lens.

### 2.4. Device Fabrication and Photodetection

First, 30 nm thick gold and 5 nm thick chromium films were deposited on RGO and MoS_2_@RGO nanoscrolls, respectively, by thermal evaporation with a TEM grid (200 mesh) as a mask.

A probe station (model TTPX, Lake Shore Inc., Rhinelander, WI, USA) and Keithley 4200 semiconductor characterization system (Advanced Test Equipment Corp., San Diego, CA, USA) were used to monitor the real-time current change of the as-prepared devices. The photocurrent was collected from individual ROG and MoS_2_@RGO nanoscrolls with Au pads as the source and drain electrodes, respectively, as shown in Figure S1. The photo-detection test was recorded under blue (405 nm), green (532 nm) and red (633 nm) lasers. The power of the laser was measured with a laser power meter (Laser power meter LP1, SanWa, Okayama, Japan).

## 3. Result and Discussion

Scheme 1 shows the preparation of MoS_2_@RGONSs by molecular combing and thermal annealing. After the (NH_4_)_2_MoS_4_ was wrapped into GO nanoscrolls by molecular combing, the (NH_4_)_2_MoS_4_ was decomposed to MoS_2_ at a high temperature under the atmosphere of N_2_/H_2_ [30]. It was found that the (NH_4_)_2_MoS_4_ was decomposed to MoS_3_ as the temperature increased from 120 to 260 °C under an inert atmosphere. The MoS_3_ was further reduced to MoS_2_ as the temperature was higher than 230 °C under N_2_/H_2_ [30]. The process can be described as the following chemical reactions,

(NH_4_)_2_MoS_4_ → 2NH_3_ + H_2_S + MoS_3_ (120–260 °C) MoS_3_ + H_2_ → MoS_2_ + H_2_S (230–450 °C) (1)

We heated the (NH_4_)_2_MoS_4_@GONSs in the temperature range of 300 to 500 °C. The Raman spectroscopy was used to characterize the amount of MoS_2_ in GONSs by measuring the peak intensity of A_1g_. As shown in Figure S2a, the peak intensity of A_1g_ increased as the temperature increased from 300 to 400 °C, while it decreased as the temperature further increased to 500 °C. The flow ratio of N_2_ to H_2_ also affects the formation of MoS_2_ during the thermal annealing process. As shown in Figure S2b, the A_1g_ peak of the (NH_4_)_2_MoS_4_@GONS annealed with a gas stream of 80 sccm N_2_ and 40 sccm H_2_ showed the highest intensity. Therefore, a mixture gas of N_2_/H_2_ (80/40 sccm) was introduced as a protective gas during the experiment. The concentration of the (NH_4_)_2_MoS_4_ solution also has an important effect on the formation of (NH_4_)_2_MoS_4_@GONSs and MoS_2_@RGONSs. The peak intensity of A_1g_ increased as the concentration of (NH_4_)_2_MoS_4_ solution increased from 5 mM to 30 mM, while it decreased when 50 mM (NH_4_)_2_MoS_4_ solution was used (Figure S2c). In addition, the diameter of the (NH_4_)_2_MoS_4_@GONSs increased as the concentration of (NH_4_)_2_MoS_4_ increased from 5 mM to 50 mM (Figure S3). Meanwhile, the long and straight nanoscrolls were changed to irregular and thick aggregations. In order to wrap more MoS_2_ and maintain the good scroll structure, (NH_4_)_2_MoS_4_ solution with a concentration of 30 mM was used as the optimal concentration and annealed at 400 °C. 

**Figure 1:**
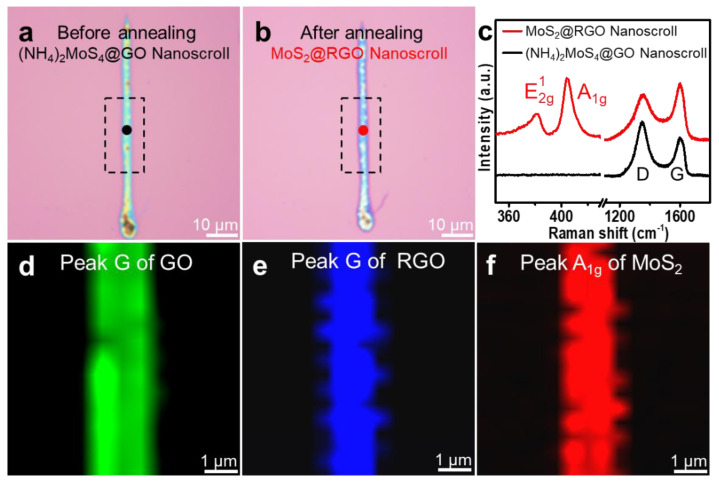
The OM images of the (NH_4_)_2_MoS_4_@GO nanoscroll (**a**) before and (**b**) after thermal annealing. (**c**) Raman spectra of the (NH_4_)_2_MoS_4_@GO and MoS_2_@RGO nanoscrolls. The Raman mapping images of (**d**) the (NH_4_)_2_MoS_4_@GO nanoscroll and (**e**) the RGO at peak G (1587 cm^−1^), and the MoS_2_@RGO nanoscroll at peak (**f**) A_1g_ (404.2 cm^−1^) of MoS_2_.

Figure 1a,b show the OM images of an (NH_4_)_2_MoS_4_@GO nanoscroll before and after thermal annealing, respectively. It can be seen that the color of the nanoscroll changed from cyan to gray blue after thermal annealing. In addition, the height of the MoS_2_@RGO nanoscroll is quite smaller than that of the (NH_4_)_2_MoS_4_@GO nanoscrolls. As shown in Figure S4, the height of the (NH_4_)_2_MoS_4_@GO is 201.4 nm, while it decreases largely to 112.5 nm after high temperature annealing, which could be attributed to the evaporation of water molecules trapped in the nanoscroll and the decomposition of (NH_4_)_2_MoS_4_. In order to confirm the formation of the MoS_2_@RGO nanoscroll, Raman spectroscopy was conducted at the same position of the nanoscroll before and after annealing. As shown in Figure 1c, there are only two Raman peaks located at 1359 and 1587 cm^−1^ for the (NH_4_)_2_MoS_4_@GO nanoscroll, which are assigned to the D and G peaks of GO. After thermal annealing, there are two more peaks located at 384.4 and 404.2 cm^−1^ besides the D and G peaks, which are characteristics of MoS_2_ nanosheets [31,32]. In addition, the intensity ratio of the D to G peak decreased from 1.24 to 0.72 for the (NH_4_)_2_MoS_4_@GO nanoscroll after thermal annealing, indicating the formation of reduced GO (RGO). The D band originates from the lattice destruction of sp^2^-hybridized carbon, and the G band arises from the first-order scattering of the $E_{2g}^{1}$ mode. The intensity ratio (I_D_/I_G_) reflects the disorder of the carbon structure, and the higher intensity ratio of I_D_/I_G_ means more defective graphitic structures. Therefore, the Raman characterization confirms the successful conversion of the (NH_4_)_2_MoS_4_@GO nanoscroll to the MoS_2_@RGO nanoscroll after thermal annealing at 400 °C for 60 min. In order to investigate whether the MoS_2_ was uniformly wrapped into the RGO nanoscroll, the as-obtained MoS_2_@RGO nanoscroll was characterized by Raman mapping. As shown in Figure 1d,e, the G peak of GO nanoscroll was unchanged after thermal annealing. Meanwhile, the Raman mapping of the A_1g_ peak of MoS_2_ showed a homogeneous signal (Figure 1f), indicating that the MoS_2_ was uniformly distributed in the RGO nanoscroll.

**Figure 2:**
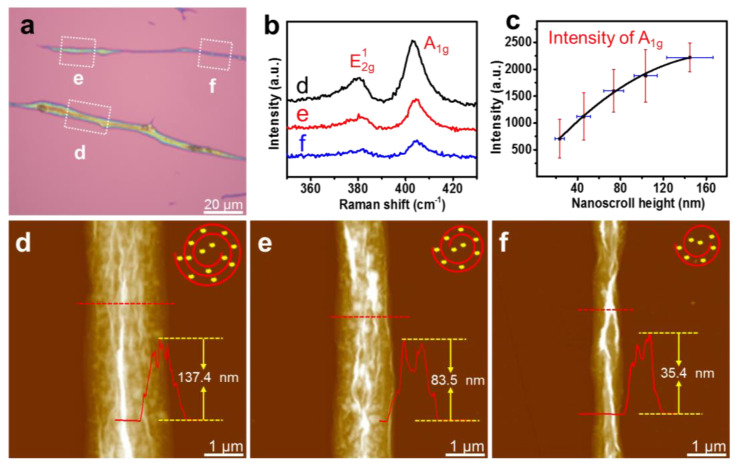
(**a**) The OM image of the MoS_2_@RGO nanoscrolls. (**b**) The Raman spectra of MoS_2_@RGO marked by dashed boxes of d, e and f shown in (**a**). (**c**) The plot of the Raman intensity of A_1g_ peak as a function of the height of the MoS_2_@RGO nanoscrolls. (**d**–**f**) The corresponding AFM images of the MoS_2_@RGO nanoscroll marked by dashed boxes of d, e and f shown in (**a**).

We found that the Raman peak intensity of MoS_2_ in the MoS_2_@RGO nanoscrolls varied with the height of the nanoscrolls. To investigate the relationship between the height of the MoS_2_@RGO nanoscroll and the amount of trapped MoS_2_ in it, the MoS_2_@RGO nanoscrolls with various heights were characterized using AFM and Raman spectroscopy, respectively. Figure 2a shows the OM image of the MoS_2_@RGO nanoscroll, where the boxes marked by d, e and f are three nanoscrolls with different heights. Figure 2d–f show the corresponding AFM images, and the measured heights of the three nanoscrolls are 137.4 nm, 83.5 nm and 35.4 nm, respectively. As shown in Figure 2b, the nanoscroll with a height of 137.4 nm presents a stronger Raman signal (box d), while the nanoscroll with a height of 35.4 nm has a weaker Raman signal. To further reveal the influence of the height of the MoS_2_@RGO nanoscrolls on the Raman peak intensity of MoS_2_, a lot of nanoscrolls were measured to plot the Raman peak intensity as a function of the height of the nanoscrolls. As shown in Figure 2c, with the increasing height of the MoS_2_@RGO nanoscrolls, the Raman peak intensity of MoS_2_ gradually increases, indicating that more MoS_2_ was trapped in a higher nanoscroll.

**Figure 3:**
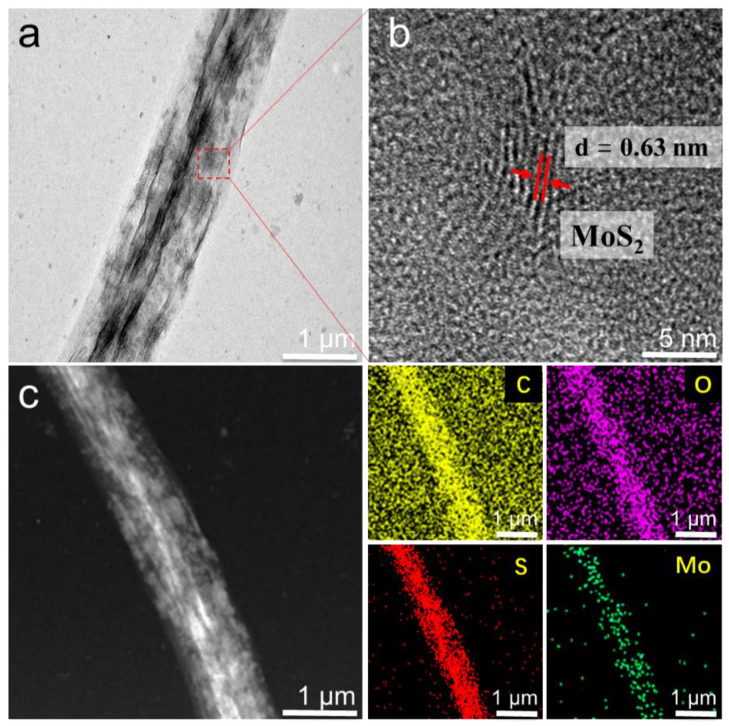
(**a**) The TEM image of the MoS_2_@RGO nanoscroll. (**b**) HRTEM image of the MoS_2_ nanosheets marked by the dashed box shown in (**a**). (**c**) The STEM image and EDX elemental mapping of the MoS_2_@RGO nanoscroll.

In order to clearly observe the detailed structure and confirm the formation of MoS_2_ in the as-prepared MoS_2_@RGO nanoscroll, high-resolution transmission electron microscopy (HRTEM) was used for characterization. Figure 3a shows the low-resolution TEM image of the MoS_2_@RGO nanoscroll. We can see that the MoS_2_@RGO nanoscroll exhibits a multilayer scrolled structure with a dark, dense inner layer. Figure 3b shows the HRTEM characterization result of the red dashed box shown in Figure 3a. The well resolved lattice stripes with spacing of 0.63 nm are clearly presented, which is consistent with the interlayer spacing of layered MoS_2_ [33]. Moreover, the energy dispersive X-ray (EDX) elemental mapping analysis of the MoS_2_@RGO nanoscroll shown in Figure 3c provides strong evidence for the homogeneous distribution of C, O, Mo and S elements in the MoS_2_@RGO nanoscroll. The HRTEM and EDX results confirm the uniform existence of the MoS_2_ nanosheet in the RGO nanoscroll.

Graphene and its derivatives are severely limited in optoelectronic applications due to their zero band gap and poor absorption of visible light. In order to improve the optoelectronic performance of graphene, MoS_2,_ as a typical transition metal dichalcogenides (TMDCs) material, has been widely used to combine graphene for photodetection [34]. By wrapping MoS_2_ into the RGO nanoscrolls, we found that the MoS_2_@RGO nanoscroll also showed promising photodetection performance. Photodetectors based on RGO nanoscrolls and MoS_2_@RGO nanoscrolls were fabricated to investigate the effect of wrapped MoS_2_. It is well known that photosensitivity is an important parameter to evaluate the performance of photodetectors [35,36,37], which is usually defined by the ratio of photocurrent to dark current (PDR), as follows:

PDR = I_photo_/I_dark_ (I_photo_: photocurrent; I_dark_: dark current) (2)

**Figure 4:**
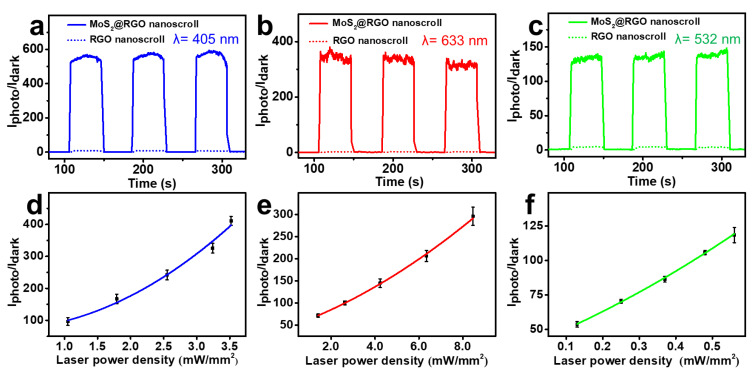
(**a**–**c**) The PDRs of photodetectors based on RGO and MoS_2_@RGO nanoscrolls under (**a**) blue, (**b**) red and (**c**) green lasers. (**d**–**f**) Plots of PDR values of photodetectors based on MoS_2_@RGO nanoscrolls under (**d**) blue, (**e**) red and (**f**) green lasers as a function of power density.

The photocurrent and dark current of the RGO nanoscrolls and MoS_2_@RGO nanoscrolls-based photodetectors were firstly measured under the dark and the illumination of blue, red and green lasers with different laser power densities, respectively. Because of the low light absorption of RGO, we found that the RGO-based photodetectors exhibited photosensitivity of ~7, ~2.4 and ~4 under blue, red and green lasers (Figure S5). Figure 4a–c show the PDRs of photodetectors based on RGO nanoscrolls and MoS_2_@RGO nanoscrolls under blue (405 nm), red (633 nm) and green (532 nm) lasers. The PDRs of photodetectors based on the MoS_2_@RGO nanoscrolls were 570, 360 and 140 under blue, red and green lasers, which are almost 81, 144 and 35 times those of the photodetectors based on the RGO nanoscrolls measured under the same conditions. In addition, the photocurrent of the MoS_2_@RGO nanoscroll is highly dependent on the power density of the incident light. As shown in Figure 4d–f, the PDRs increased as the incident laser power density increased. The different photoresponse of MoS_2_@RGONS to blue, green and red light could be explained as follows. In our experiment, the power intensity of green light is the lowest. However, the PDR of MoS_2_@RGONS is around 140 at a power density of 0.56 mW/mm^2^ (Figure 4f), while the PDRs of MoS_2_@RGONS are around 100 and 70 for blue and red lasers at power densities of 1.05 mW/mm^2^ and 1.41 mW/mm^2^ (Figure 4d,e), respectively. The MoS_2_ nanosheets synthesized in GONS could be multilayer, which can be confirmed by the HRTEM images shown in Figure 3b. Therefore, the multilayer MoS_2_ trapped into RGONS should be more suitable for detecting green lasers than blue and red lasers given that they are at the same power intensity. A similar phenomenon has also been reported in a multilayer MoS_2_@glassy-graphene heterostructure [38]. In addition, the MoS_2_@RGONS shows higher PDR under the blue laser than under the red laser at similar power intensities. This could be attributed the higher photon energy of the blue laser compared to the red laser, which can generate more photoinduced carriers at the same power [39].

The excellent photodetection performance of the MoS_2_@RGO nanoscroll could be attributed to the formation of multiple heterojunction interfaces between the RGO and MoS_2_ nanosheets. It is known that the ultrafast separation and transfer of photogenerated carriers can be achieved at the heterojunction interface of graphene and MoS_2_, resulting in a substantial increase in photocurrent and photoresponse. Due to the roll-up structure of the MoS_2_@RGO nanoscroll, the MoS_2_ nanosheets are wrapped between adjacent RGO layers spirally, forming multiple heterojunction interfaces. When light was shined on the MoS_2_@RGO nanoscroll, the MoS_2_ nanosheets in each heterojunction interface could absorb light, and charge carriers were generated simultaneously. Meanwhile, the photogenerated charge carriers can be separated and transferred in an ultrafast way. Therefore, the photocurrent of the MoS_2_@RGO nanoscroll can be greatly enhanced due to the synergetic enhancement of photocurrent at each heterojunction interface. As a consequence, the photosensitivity of the MoS_2_@RGO nanoscroll is much higher than that of the RGO nanoscroll.

## 4. Conclusions

In summary, (NH_4_)_2_MoS_4_ was encapsulated into the GO nanoscrolls by the molecular combing method on hydrophobic substrate. By optimizing the precursor concentration and annealing temperature, the (NH_4_)_2_MoS_4_ and GO nanoscrolls were successfully converted to MoS_2_ and RGO nanoscrolls, forming the MoS_2_@RGO nanoscroll. The OM and AFM characterization results showed that the high-density MoS_2_@RGO nanoscrolls were successfully prepared. The uniform distribution of the MoS_2_ nanosheets in the RGO nanoscrolls was confirmed by the Raman spectroscopy and HRTEM characterization. Compared to the RGO nanoscroll, the MoS_2_@RGO nanoscroll showed much better photodetection performance. The PDRs of photodetectors based on the MoS_2_@RGO nanoscrolls were about two orders of magnitude higher than those of photodetectors based on the RGO nanoscrolls under blue, red and green lasers. The formation of multiple graphene/MoS_2_ heterojunction interfaces in a scrolled structure can not only enhance the light absorption of MoS_2_ but also accelerate the electron-hole separation. Our work indicates that the MoS_2_@RGO nanoscrolls could be promising materials for high-performance graphene-based photodetectors.

## Data Availability

Data can be available upon request from the authors.

## Supplementary Materials

The following supporting information can be downloaded at https://www.mdpi.com/article/10.3390/nano12091581/s1: Figure S1. The optical images of photodetectors based on individual (a) RGO nanoscroll and (b) MoS_2_@RGO nanoscroll with Au pads as source and drain electrodes. Figure S2. Plots of Raman peak intensity of A_1g_ as function of (a) annealing temperature, (b) the flow ratio of N_2_ to H_2_, and (c) concentration of (NH_4_)_2_MoS_4_. Figure S3. OM images of (NH_4_)_2_MoS_4_@GONS prepared by molecular combing (NH_4_)_2_MoS_4_ solution with concentration of (a,e) 0.005 M, (b,f) 0.01 M, (c,g) 0.03 M, and (d,h) 0.05 M before and after thermal annealing. Figure S4. AFM height images of the same (NH_4_)_2_MoS_4_@GONS (a) before and (b) after thermal annealing. Figure S5. PDR plots of RGO nanoscrolls measured under (a) blue, (b) red, and (c) green lasers.
