# Supplementary material for: Direct Synthesis of MoS2 Nanosheets in Reduced Graphene Oxide Nanoscroll for Enhanced Photodetection
Source: Nanomaterials (Basel). 2022 May 6;12(9):1581. doi: 10.3390/nano12091581 (PMC9101584; doi:10.3390/nano12091581)
Supplement: Supplementary file 1 [file nanomaterials-12-01581-s001.zip › nanomaterials-1706424-supplementary.pdf]

nanomaterials

MDPI

Supplementary Materials

Direct Synthesis of MoS_2 Nanosheets in Reduced Graphene Oxide Nanoscroll for Enhanced Photodetection

Zhikang Wu, Feifei Li, Xiya Li, Yang Yang, Xiao Huang * and Hai Li *

Key Laboratory of Flexible Electronics (KLOFE), Institute of Advanced Materials (IAM), Nanjing Tech University (Nanjing Tech), 30 South Puzhu Road, Nanjing 211816, China; iamzkwu@njtech.edu.cn (Z.W.); lifeifei@njtech.edu.cn (F.L.); lixiya@njtech.edu.cn (X.L.); iamyyang@njtech.edu.cn (Y.Y.)

* Correspondence: iamxhuang@njtech.edu.cn (X.H.); iamhli@njtech.edu.cn (H.L.)

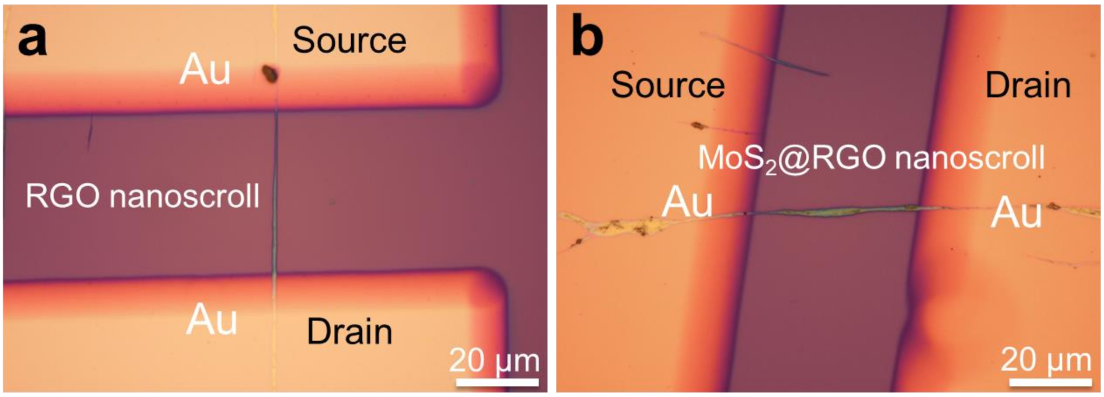

Figure S1. The optical images of photodetectors based on individual (**a**) RGO nanoscroll and (**b**) MoS_2@RGO nanoscroll with Au pads as source and drain electrodes.

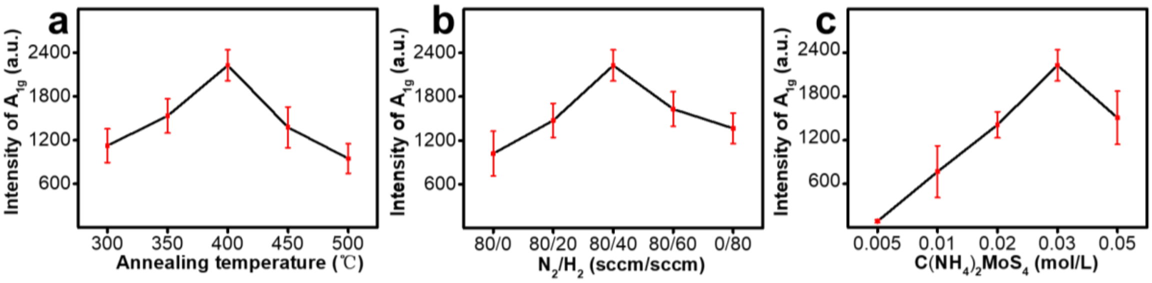

Figure S2. Plots of Raman peak intensity of A_{1g} as function of (**a**) annealing temperature, (**b**) the flow ratio of N_2 to H_2, and (**c**) concentration of $(NH_4)_2MoS_4$.

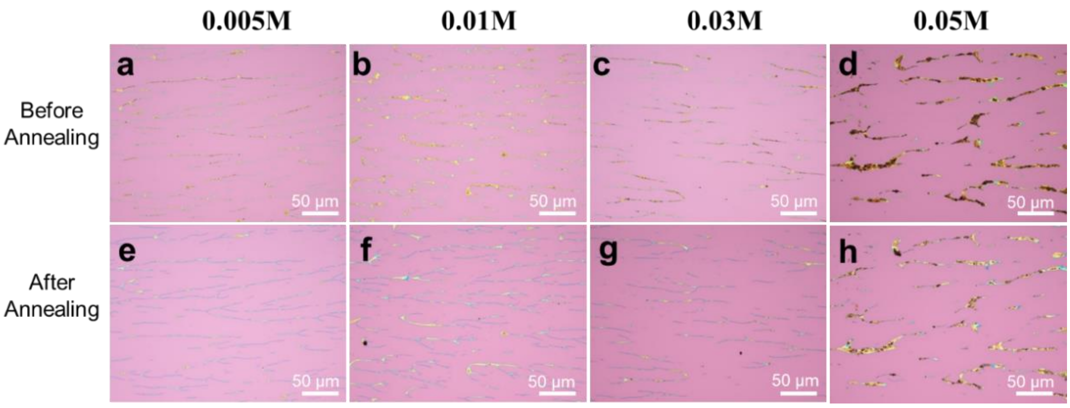

Figure S3. OM images of $(NH_4)_2MoS_4$@GONS prepared by molecular combing $(NH_4)_2MoS_4$ solution with concentration of (**a**,**e**) 0.005 M, (**b**,**f**) 0.01 M, (**c**,**g**) 0.03 M, and (**d**,**h**) 0.05 M before and after thermal annealing.

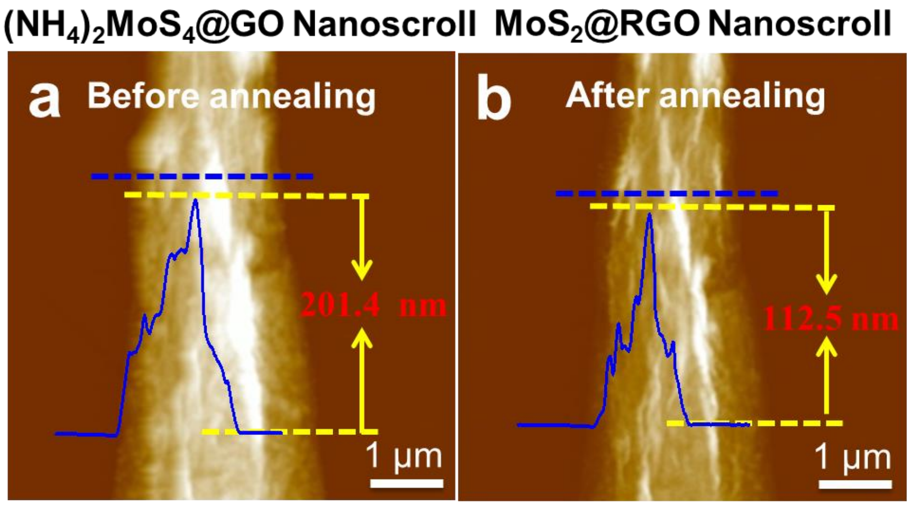

Figure S4. AFM height images of the same $(NH_4)_2MoS_4$@GONS (**a**) before and (**b**) after thermal annealing.

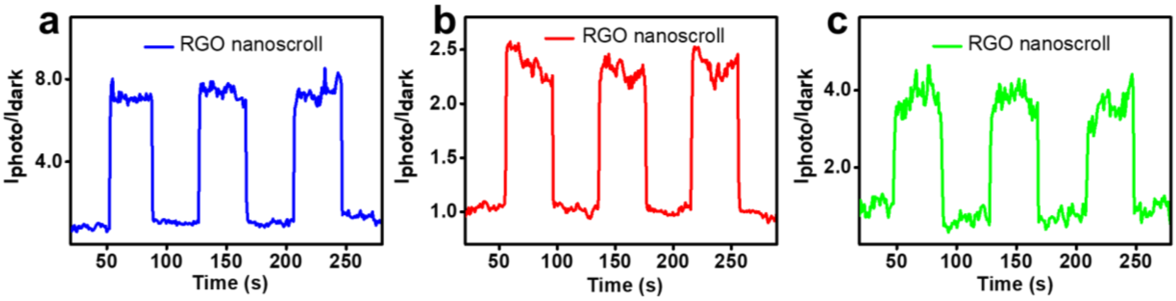

Figure S5. PDR plots of RGO nanoscrolls measured under (**a**) blue, (**b**) red, and (**c**) green lasers.
